# Silencing the alternative

**DOI:** 10.7554/eLife.49635

**Published:** 2019-08-06

**Authors:** Priya Sivaramakrishnan, John Isaac Murray

**Affiliations:** Department of GeneticsPerelman School of MedicinePhiladelphiaUnited States

**Keywords:** ZTF-11, Myt1, neurogenesis, MuvB complex, transcriptional repression, neuronal differentiation, *C. elegans*

## Abstract

The transcription factor *ztf-11* promotes neuronal differentiation by repressing other cell fates in the nematode worm *C. elegans*.

**Related research article** Lee J, Taylor CA, Barnes KM, Shen A, Stewart EV, Chen A, Xiang YK, Bao Z, Shen K. 2019. A Myt1 family transcription factor defines neuronal fate by repressing non-neuronal genes. *eLife* **8**:e46703. doi: 10.7554/eLife.46703

During development, cells have to divide and differentiate to generate the various types of cells found in the adult organism. To do this, cells must activate the genes necessary to become the right type of cell, but also repress the genes that would drive them to different fates. Many of the transcription factors that activate gene expression during differentiation have been studied extensively, but only a few examples of transcription factors that repress unwanted cell fates have been described (Bellefroid et al., 1996; Vasconcelos and Castro, 2017).

Transcription factors are also important for cell fate reprogramming, a process through which an adult cell can lose its identity and become a new cell type. In vitro, this is usually done by forcing the expression of specific transcription factors.

The Myt1 family of transcription factors are specifically expressed in vertebrate neurons and are thought to repress non-neural fates. Myt1 proteins promote the formation of neurons (neurogenesis) in vertebrate embryos by blocking Notch signaling (Bellefroid et al., 1996; Vasconcelos et al., 2016). Forcing expression of Myt1l in cultured connective tissue cells called fibroblasts blocks the Notch pathway and represses non-neuronal genes, reprogramming these cells into neurons (Vierbuchen et al., 2010; Mall et al., 2017). It is not known whether Myt1 proteins repress a broad range of genes during in vivo development, partly because interpreting Myt1 loss of function in mammalian embryos is complicated by other related proteins that may have similar effects.

Now, in eLife, Kang Shen of Stanford University and co-workers – including Joo Lee as first author – report that *ztf-11*, the only member of the Myt1 family found in the nematode worm *Caenorhabditis elegans*, promotes neuronal differentiation by blocking other fates (Lee et al., 2019). All *C. elegans* individuals contain the same number of neuronal progenitors and neurons, providing a powerful framework to understand the process of neurogenesis (Sulston et al., 1983). Moreover, neurons formed during embryonic development in *C. elegans* are produced by undifferentiated progenitor cells (cells that can give rise to any cell type), whereas those formed in the larval stages are produced by skin cell precursors (Sulston and Horvitz, 1977). Lee et al. show that, like Myt1 proteins in mammals, *ztf-11* is broadly expressed in both embryonic and larval neuronal progenitors.

First, Lee et al. investigate a natural reprogramming event during which a differentiated epithelial cell is transformed into a motor neuron (Jarriault et al., 2008). It is shown that *ztf-11* is required to turn off epithelial gene expression in this reprogramming event. Next, it is shown that *ztf-11* is also necessary to turn off epithelial gene expression during other neuronal differentiations in the larva, including the production of sensory neurons and glia from another epidermal cell.

Lee et al. also find that forcing expression of *ztf-11* in epidermal cells that do not normally produce neurons was sufficient for these cells to lose epithelial identity and start to look and behave like neurons. This finding suggests that in absence of other epidermal-promoting factors, these epidermal cells would become neural cells. This is reminiscent of the hypothesis that the precursors of brain and skin cells in vertebrates have a neural ground state, which is inhibited by signaling molecules in epidermal cells (Hemmati-Brivanlou and Melton, 1997). Future work will reveal if a similar reprogramming by Myt1 proteins occurs in other systems.

Lee et al. then investigate the role of *ztf-11* in Notch signaling. In vertebrates Myt1 appears to act by repressing the Notch target gene Hes1 (Vasconcelos and Castro, 2017). In the worm, however, *ztf-11* is not required to repress the gene *lin-22*, which is the homolog of Hes1 in *C. elegans*. Rather, the regulation seems to go in the opposite direction, with *lin-22* repressing a gene called *lin-32/Atonal*, which is an activator of *ztf-11*.

Together, these results show the parallels between *ztf-11* and Myt1l-dependent repression of non-neuronal genes during fibroblast to neuron reprogramming (Mall et al., 2017), suggesting that similar pathways operate during in vitro and in vivo reprogramming.

Genome-wide expression changes in *ztf-11* mutant embryos were measured with RNA-seq and suggest that *ztf-11* is required in the embryo to fully repress both epithelial and muscle-specific genes, although it is unclear in which cells this regulation occurs. Worms mutant for *ztf-11* have a lethal paralysis phenotype, and consistent with this Lee et al. find that progenitors of neurons in the embryo express *ztf-11*. Surprisingly, they also find that in the absence of *ztf-11* a roughly normal number of neurons are generated.

A *ztf-11* reporter was used to show that the gene is broadly expressed in early embryonic precursors that are fated to produce multiple cell types, and then lost from cells that do not produce any neurons. This differs from the situation in larvae, where *ztf-11* is not expressed in progenitors but becomes activated specifically in cells that later become neurons. This suggests that the expression of *ztf-11* in the embryo might prevent epithelial and muscle genes from being expressed too early (Figure 1A). In contrast to vertebrate Myt1, *ztf-11* expression appears to be lost from most embryonic and larval neurons once they differentiate. This suggests that, at least in *C. elegans*, the role of *ztf-11* may be to establish initial repression of inappropriate lineage genes, and that this repression is then maintained by other mechanisms (Figure 1B).

**Figure 1.**
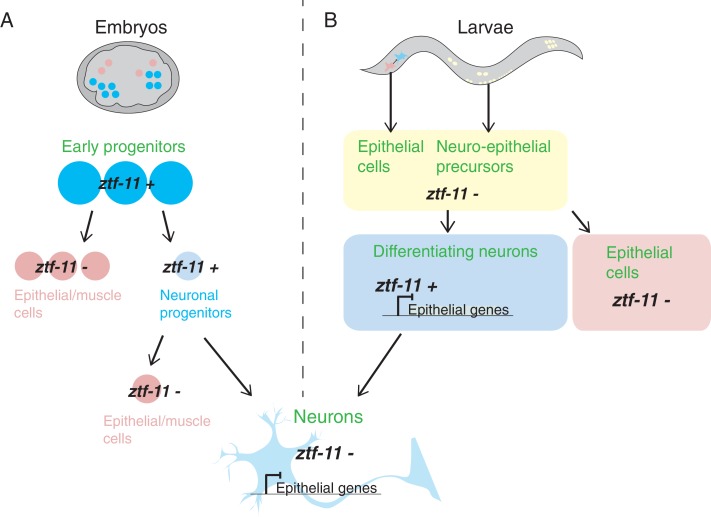
Expression of *ztf-11* during neuronal development in *C.elegans* embryos and larvae. (**A**) In *C. elegans* embryos, *ztf-11* is expressed broadly in early progenitors (*ztf-11*+). These early progenitors subsequently differentiate to produce neuronal progenitors (in which expression of *ztf-11* is maintained, thus repressing epithelial and muscle fates) and epithelial/muscle cells (which do not express *ztf-11*). The neuronal progenitors can differentiate to produce neurons or other cell types such as epithelial or muscle cells. Once neurons fully differentiate (pale blue at the bottom of the figure), they no longer express *ztf-11*. The mechanisms responsible for the repression of epithelial and muscle fates in these cells are not understood. (**B**) In *C. elegans* larvae, *ztf-11* is not initially present in neuro-epithelial precursors or epithelial cells that eventually transdifferentiate into neurons. *ztf-11* is turned on by pro-neural factors in differentiating neurons, where it represses epithelial fates. Differentiated neurons lack *ztf-11* suggesting that other unknown factors take on the role of repression (bottom image).

Image credit: P Sivaramakrishnan and J I Murray

Further studies will be needed to identify these mechanisms, and to determine if similar broad transcriptional repressors exist for other cell types. This study is a significant step forward in our understanding of the repression mechanisms that restrict some but permit other fates to be established during embryogenesis.
